# In Vitro Uptake of Hydroxyapatite Nanoparticles and Their Effect on Osteogenic Differentiation of Human Mesenchymal Stem Cells

**DOI:** 10.1155/2018/2036176

**Published:** 2018-06-19

**Authors:** Xing Yang, Yuanyuan Li, Xujie Liu, Ranran Zhang, Qingling Feng

**Affiliations:** ^1^State Key Laboratory of New Ceramics and Fine Processing, School of Materials Science and Engineering, Tsinghua University, Beijing 100084, China; ^2^Department of Stomatology, Shengli Oilfield Central Hospital, Dongying 257034, China; ^3^Graduate School at Shenzhen, Tsinghua University, Shenzhen 518055, China; ^4^Key Laboratory of Advanced Materials of Ministry of Education of China, School of Materials Science and Engineering, Tsinghua University, Beijing 100084, China

## Abstract

There have been many applications in biomedical fields based on hydroxyapatite nanoparticles (HA NPs) over the past decades. However, the biocompatibility of HANPs is affected by exposure dose, particle size, and the way of contact with cells. The objective of this study is to investigate the effect of HA NPs with different sizes on osteogenesis using human mesenchymal stem cells (hMSCs). Three different-sized HA NPs (~50, ~100, and ~150 nm, resp.) were synthesized to study the cytotoxicity, cellular uptake, and effect on osteogenic differentiation of hMSCs. The results clearly showed that each size of HA NPs had dose-dependent cytotoxicity on hMSCs. It was found that HA NPs could be uptaken into hMSCs. The osteogenic differentiation of hMSCs was evaluated through alkaline phosphatase (ALP) activity measurement, ALP staining, immunofluorescent staining for osteopontin (OPN), and real-time polymerase chain reaction (RT-PCR) examination. As expected, HA NPs of all sizes could promote the differentiation of hMSCs towards osteoblast lineage. Among the three sizes, smaller-sized HA NPs (~50 and ~100 nm) appeared to be more effective in stimulating osteogenic differentiation of hMSCs.

## 1. Introduction

Over the past few decades, nanotechnology and nanoscience have been emerging with the rise in manufacture of various nanomaterials [1]. Recently, biomaterials based on nanoparticles have become a very popular research field for applications in biomedicine, tissue engineering, and health care system [2–4]. Compared with traditional medicine, nanoparticles exhibit new possibilities for many technological applications [5]. Hydroxyapatite (HA) with chemical formula of Ca_10_(PO_4_)_6_(OH)_2_ is the major mineral constituent of human hard tissue (bones and teeth) [6]. Owing to the excellent biocompatibility [7], hydroxyapatite nanoparticles (HA NPs) play an important role in various biomedical applications. For instance, HA NPs can be used for bioimaging, photodynamic therapy, gene/drug delivery, and hard tissue repair [8–12]. In order to meet the requirements of various applications in the biomedical field, HA NPs with different sizes and aspect ratios have been prepared by using surfactant molecules as structure-directing agents [13–15]. These surfactants are normally toxic for biomedical applications. To remove the cytotoxic surfactants, several methods have been proposed [16, 17]. For example, calcination is a common strategy for removing surfactant molecules from HA NPs, which will change their morphology and size distribution [16], while the reaction-dissolution approach can clean the surface of HA NPs with unchanged topographic characteristics (shape and size distribution) and improved biocompatibility [17].

Many researchers have focused on the potential of HA NPs used for bone repair and regeneration. In a previous study, Wang et al. prepared the HA NPs/polyamide composite scaffolds and found that the scaffolds had no negative effects on osteogenic differentiation of rabbit bone marrow-derived mesenchymal stem cells [18]. He et al. prepared micropatterns composed of HA NPs, and osteoblasts were proven to be well localized on the HA microislands [19]. In addition, HA NPs were not only used for investigation of osteogenic differentiation in vitro but also bone defect repair *in vivo* as well. Liu et al. confirmed the osteoinductivity of HA NPs/silk fibroin composite scaffolds through both cell experiment *in vitro* and calvarial defect model *in vivo* [20]. Zhang et al. found that 3D porous scaffold composed of HA NPs (surface grafted with PLLA) and PLGA enhanced the *in vivo* mineralization and osteogenesis through investigation of intramuscular implantation and repairing radius defects of rabbits [21]. Similarly, the HA NP-coated PLGA scaffolds have exhibited improved biocompatibility and facilitated the bone defect restoration of rabbits [22].

As noted above, HA NPs have been a very attractive material used in bone repair and regeneration because of appropriate physicochemical properties and biological characteristics. However, previous researches involving HA NPs and osteogenesis mainly focused on the influence of coatings or scaffolds incorporated with HA NPs on osteogenic differentiation of bone-related cells. On the other hand, due to the different physicochemical characteristics and bioactivity from bulk materials, HA NPs have also been applied for a number of applications in biomedicine, such as drug delivery, transfection, and gene silencing [8]. Among these applications, HA NPs interacted directly with the related cells and then were internalized into the cells. Due to the internalization of HA NPs, cells incubated with HA NPs directly may show different responses compared with that cultured on the surface of HA NPs containing coatings or scaffolds. Some recent studies showed that the biocompatibility of HA NPs varied as exposure dose, particle size, and the way of contact with cells [1, 23–25]. There is a lack of information concerning the bioactivity in the human tissue cells as it relates to the size of HA NPs. Therefore, it is of great interest to investigate the effect of internalization of different-sized HA NPs on human tissue cells. Understanding the cell response to different-sized HA NPs is beneficial to choose the effective candidates for bone tissue regeneration.

In the bone tissue, hMSCs play a key role because they are multipotent cells which can renew themselves and differentiate into osteoblast [26, 27]. Osteogenesis is a part of bone cellular metabolism, which is essential to bone remodeling [28]. The hMSCs are usually applied as seed cells for bone tissue engineering. For these reasons, hMSCs, derived from bone marrow, are one of the best *in vitro* model systems for investigating the osteogenesis potential of nanobiomaterials in human.

Hence, the aim of this study is to evaluate the in vitro uptake of HA NPs with different sizes and their effect on osteogenic differentiation of bone marrow-derived hMSCs. Three different-sized HA NPs were prepared by chemical precipitation method. The hMSCs were exposed to different concentrations of HA NPs for 24 h and then induced to osteogenic differentiation. The potential effects of these HA NPs on cell viability and expression of several osteogenic were analyzed.

## 2. Materials and Methods

### 2.1. Preparation and Characterization of HA NPs

HA NPs with three different sizes (designated herein as S50, S100, and S150, resp.) were prepared by the conventional chemical precipitation [6, 29]. In brief, calcium chloride (CaCl_2_) solution was dropwise added to diammonium hydrogen phosphate ((NH_4_)_2_HPO4) solution under continuous and gentle stirring, while the molar ratio of Ca/P was kept at 1.67. During the precipitation, aqueous ammonia was added to adjust the pH value to 7 for S50, 8 for S100, and 10 for S150. The temperature was maintained at 30°C for S50, 50°C for S100, and 90°C for S150. After precipitation, the resultant suspension was aged at room temperature for 16 h. Finally, the resulting powders were collected, rinsed with deionized water, and dried by vacuum freeze drying.

The morphology and size of the synthesized powders were characterized by transmission electron microscope (TEM, Tecnai G20, USA). The crystalline phases of the synthesized powders were determined using X-ray diffraction (XRD, Rigaku D/max-2500 PC, Japan). The Brunauer–Emmett–Teller (BET) surface area was measured by Autosorb-IQ2-MP (Quantachrome Instruments, USA). The hydrodynamic diameter of S50, S100, and S150, dispersed in basal medium (Cyagen Biosciences Inc., China) with 10% fetal bovine serum, was measured by dynamic light scattering (DLS, Zetasizer Nano ZS90, UK).

### 2.2. Cell Culture

Bone marrow-derived hMSCs were cultured in a growth medium (Cyagen Biosciences Inc., USA) containing 10% fetal bovine serum (FBS), 1% glutamine, and 1% penicillin-streptomycin at 37°C in humid air containing 5% CO_2_. The growth medium was changed every 48 h.

To induce osteogenic differentiation, the culture medium was changed to osteogenic inductive medium (Cyagen Biosciences Inc., USA) after hMSCs treated with HA NPs for 24 h. The inductive medium was refreshed every 48 h.

### 2.3. Cell Viability Assay

The Cell Counting Kit-8 (CCK-8, Dojindo, Japan) assay was used to evaluate the effect of HA NPs on cell viability of hMSCs. Cells were cultured with different concentrations (0, 5, 10, and 50 *μ*g/ml) of HA NPs for 24 h at 5% CO_2_, 37°C. After 1, 3, and 5 days of osteoinduction, the cells were incubated with CCK-8 solution (consisting of 90% growth medium and 10% CCK-8) for 3 h at 37°C. Afterwards, the absorbance (OD) of the incubation solution was measured at 450 nm.

### 2.4. Cellular Uptake of HA NPs

TEM was employed to observe cellular uptake of HA NPs by hMSCs. After incubation with 10 *μ*g/ml of HA NPs for 24 h, the cells were harvested and fixed in 2.5% glutaraldehyde at 4°C overnight. Then, the cells were postfixed in 0.1 M cacodylate buffer containing 1% osmium tetroxide for 1 h at 4°C, dehydrated stepwise in ethanol, and embedded in epoxy resin. Ultrathin sections (70 nm) were cut using an ultramicrotome (Lecia, Germany), collected on copper grids, and observed by TEM (Hitachi H-7650B, Japan).

### 2.5. ALP Activity Assay

The cells were incubated with 0 and 10 *μ*g/ml HA NPs for 24 h and then induced into osteogenic differentiation. ALP activity assay was performed on days 7 and 14 after osteogenic induction. Briefly, the cells were lysed in 100 *μ*l RIPA lysis buffer (Beyotime, China). The lysate was then analyzed according to the manufacturer's protocol (ALP assay kit, Jiancheng, China). The ALP activity was normalized by the total protein content, which was measured by a bicinchoninic acid assay kit (Aidlab, China).

### 2.6. Immunofluorescent Staining for Osteopontin (OPN)

Immunofluorescent staining was applied to evaluate the expression of OPN at protein level. The cells were incubated with 0 and 10 *μ*g/ml HA NPs for 24 h and then induced into osteogenic differentiation. On day 14 of differentiation, the cells were washed with PBS, fixed with 4% paraformaldehyde, permeabilized with 0.3% Triton X-100, and blocked with 10% goat serum for 2 h. Thereafter, the cells were incubated with the rabbit polyclonal antibodies against OPN (Abcam, USA) at 4°C overnight and treated with Dylight 594-conjugated goat anti-rabbit IgG (Abbkine, USA) for 1 h. The cell nuclei were then stained with 4′,6-diamidino-2-phenylindole (DAPI) at room temperature for 15 min. Subsequently, the samples were viewed using a laser scanning confocal microscope (LSCM, Zeiss710 META, Germany).

### 2.7. Real-Time Polymerase Chain Reaction (RT-PCR)

The expression of ALP, OPN, runt-related transcription factor 2 (Runx2), and osteocalcin (OCN) at gene level were quantitatively analyzed by RT-PCR (the primers used for the RT-PCR study are shown in Table 1). The cells were incubated with 0 and 10 *μ*g/ml HA NPs for 24 h and then induced into osteogenic differentiation. On day 14 after osteogenic induction, total RNA of the cells was extracted using TRNzol Reagent (Tiangen, China). Then, 400 ng of total RNA was reverse transcribed into cDNA using FastQuant RT kit (Tiangen, China). RT-PCR was performed on T100 Thermal Cycler (BioRad, USA) using iTaq Universal SYBR Green Supermix (BioRad, USA).

### 2.8. Calcium Ion Release

The degradation experiment *in vitro* was performed by immersing 10 μg/ml of HA NPs in Dulbecco's phosphate-buffered saline (DPBS) at 37°C for 14 days. The concentration of calcium ions released by HA NPs was measured by inductively coupled plasma optical emission spectrometer (ICP-OES).

### 2.9. Medium pH Measurement

In order to investigate the effect of HA NPs on the pH of culture medium, 10 *μ*g/ml of HA NPs was immersed in the osteogenic inductive medium (Cyagen Biosciences Inc., USA) at 37°C in humid air containing 5% CO_2_. Since the osteogenic inductive medium was changed every 2 days, the pH of medium with and without HA NPs was measured after 2 days of incubation.

### 2.10. Statistical Analysis

All data were expressed as the mean ± standard deviation (SD) of three independent experiments and analyzed using one-way analysis of variance (ANOVA) by SPSS software (15.0.1). A *p* < 0.05 was considered statistically significant.

## 3. Results

### 3.1. Characterization of HA NPs

**Figure 1:**
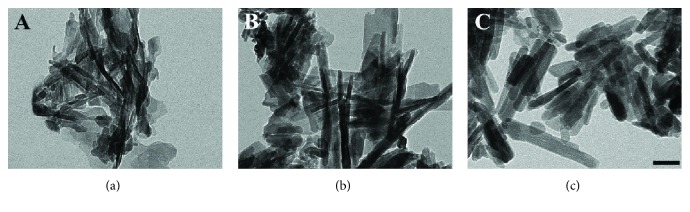
TEM images of S50 (a), S100 (b), and S150 (c). The bar is 50 nm.

Figure 1 shows the TEM images of HA NPs (S50, S100, and S150). The length of S50, S100, and S150 was approximately 50, 100, and 150 nm (Figure 1(c)), while the width was around 8, 15, and 20 nm, respectively. The hydrodynamic diameter from DLS analysis was 567.86 ± 19.71 nm for S50, 626.10 ± 14.95 nm for S100, and 1262.33 ± 46.5 nm for S150 (Table 2), which revealed that HA NPs were aggregated in the cell culture medium. As shown in Table 2, S50 and S100 showed similar specific surface area, which is much larger than that of S150.

**Figure 2:**
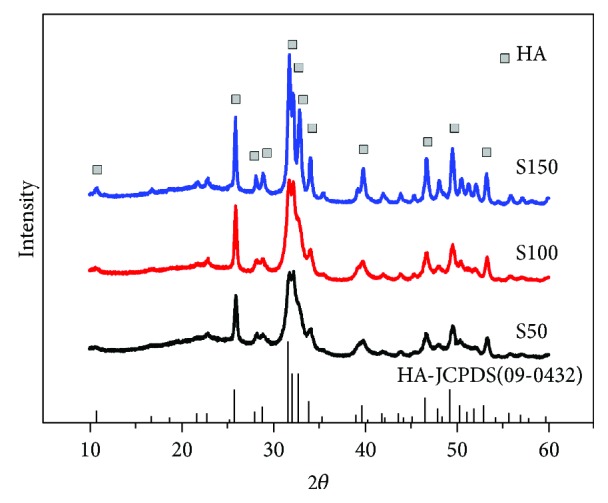
XRD patterns of S50 (black line), S100 (red line), and S150 (blue line).

XRD patterns of S50, S100, and S150 (Figure 2) display typical characteristic diffraction peaks of crystalline HA phase (25.87°, 31.78°, 46.71°, 49.47°, and 53.14°) according to the standard card of HA (JCPDS 09-0432) [30, 31]. Moreover, S50 and S100 showed broadening diffraction peaks, indicating that they consisted of poorly crystalline and small crystals. In contrast, the diffraction peaks of S150 were sharper than those of S50 and S100, implying the higher crystallinity of S150.

### 3.2. Cell Viability Assay

**Figure 3:**
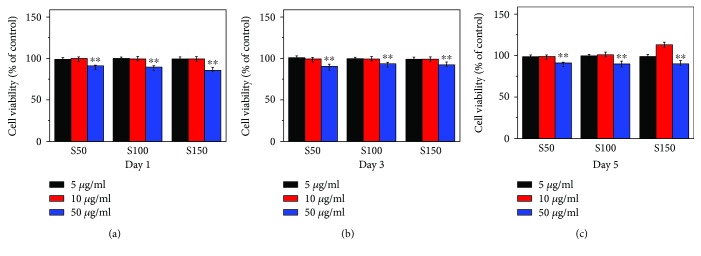
Cell viability detected by CCK-8 assay on days 1, 3, and 5 after osteoinduction. The hMSCs were treated with different concentrations (0, 5, 10, and 50 *μ*g/ml) of HA NPs for 24 h at 5% CO_2_, 37°C. Values are expressed as mean ± SD (*n* = 3 for each sample). ^∗∗^*p* < 0.01 compared to the control group (hMSCs without HA NP treatment).

To evaluate the effect of HA NPs on cell viability of hMSCs, CCK8 assay was applied on days 1, 3, and 5 after osteogenic induction. As shown in Figure 3, the cell viability of hMSCs treated with 5 and 10 *μ*g/ml of HA NPs was comparable to the control group. However, there were significant decreases in cell viability of hMSCs treated with 50 *μ*g/ml of HA NPs compared with that of the control group at each time point. The result indicated that HA NPs showed cytotoxicity to hMSCs in a concentration-dependent manner. Based on the result of cell viability assay, the maximum safety concentration, that is, 10 *μ*g/ml, was selected for subsequent experiments.

### 3.3. Cellular Uptake of HA NPs

**Figure 4:**
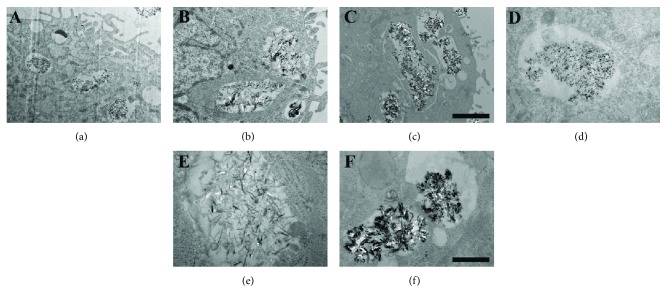
TEM images of cellular uptake of HA NPs in hMSCs. The cells were incubated with 10 *μ*g/ml of S50 (a, d), S100 (b, e), and S150 (c, f) for 24 h. The bar is 2 *μ*m for (a–c), 500 nm for (d–f).

As shown in Figure 4, S50, S100, and S150 were uptaken into hMSCs after incubation for 24 h at the concentration of 10 *μ*g/ml, and they distributed in some different-sized vesicles in cell cytoplasm. The HA NPs were irregularly aggregated, and the size of some aggregation reached several microns (Figures 4(a)–4(c)). The size and morphology of the HA NPs were further confirmed in Figures 4(d)–4(f).

### 3.4. Osteogenic Differentiation

**Figure 5:**
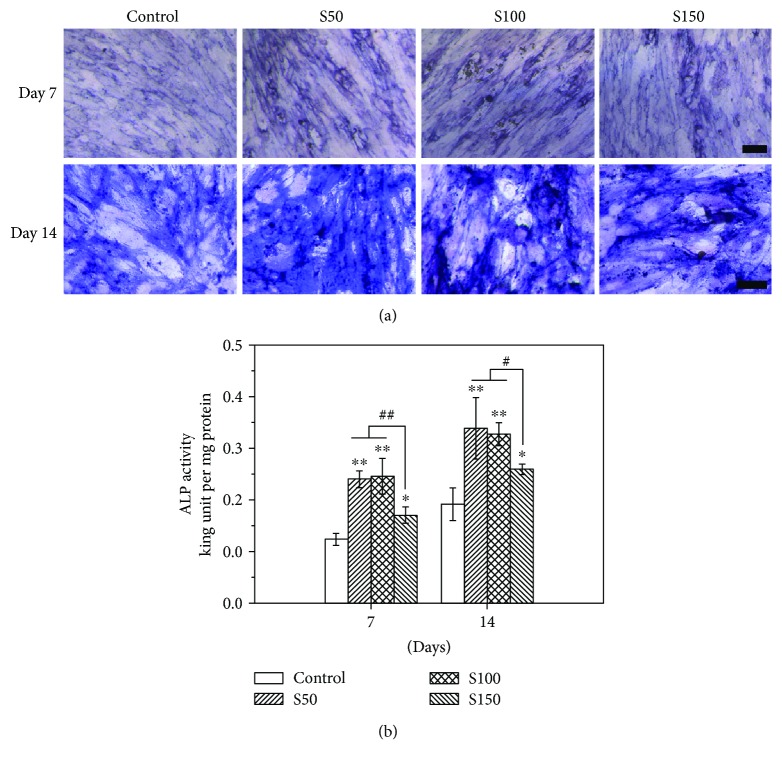
ALP staining (a) and ALP activity (b) of hMSCs after 7 and 14 days of osteoinduction. The hMSCs were incubated with 0 and 10 *μ*g/ml of HA NPs for 24 h. ^∗^*p* < 0.05, ^∗∗^*p* < 0.01 comparison between the control group (hMSCs without HA NP treatment) and other groups. ^#^*p* < 0.05, ^##^*p* < 0.01 comparison between S50 group, S100 group, and S150 group.

ALP activity of hMSCs was qualitatively and quantitatively assayed on days 7 and 14 after osteogenic induction. The ALP activity of hMSCs increased from days 7 to 14 (Figure 5). As shown in Figure 5(a), more intense color was observed for cells of HA NP treatment groups in comparison with that of the control group. Furthermore, for the cells treated with S50, S100 seemed to stain more strongly and homogenously than those treated with S150. The quantitative result (Figure 5(b)) also showed that the addition of HA NPs increased the ALP activity of hMSCs. In addition, the cells incubated with smaller HA NPs (S50 and S100) showed higher ALP activity compared with the S150 group at each time point.

**Figure 6:**
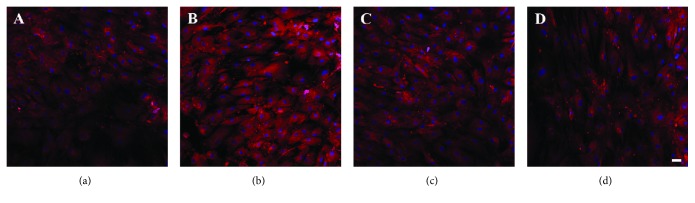
Immunofluorescent staining for OPN on day 14 after osteogenic induction (scale bar is 50 *μ*m). The cells were exposed to HA NPs for 24 h (a) 0 *μ*g/ml, (b) S50—10 *μ*g/ml, (c) S100—10 *μ*g/ml, and (d) S150—10 *μ*g/ml.

The expression of OPN at protein level was detected by immunofluorescent staining. On day 14 after osteogenic induction, the cells were strongly positive to OPN for all groups (Figure 6). Cells incubated with 10 *μ*g/ml HA NPs displayed higher fluorescence intensity compared with those of the control group. Moreover, the cells showed higher expression extent of OPN treated with S50 and S100 than those exposed to S150.

**Figure 7:**
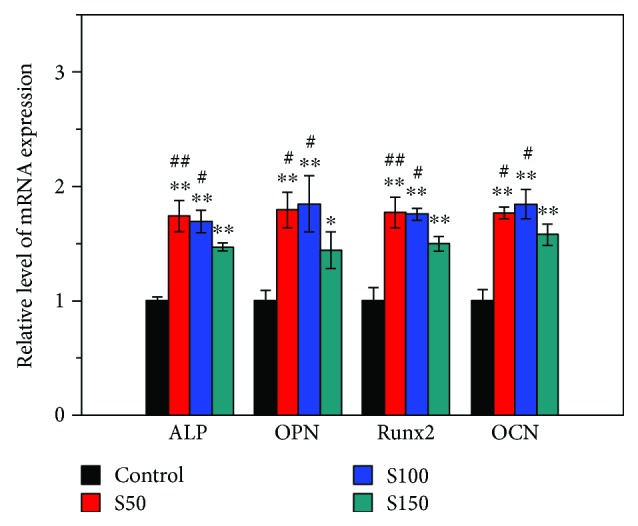
mRNA expression levels of ALP, OPN, Runx2, and OCN on day 14 after osteogenic induction. The cells were incubated with 0 and 10 *μ*g/ml HA NPs for 24 h. Values are expressed as mean ± SD (*n* = 3 for each sample). ^∗^*p* < 0.05, ^∗∗^*p* < 0.01 compared to the control group (hMSCs without HA NP treatment). ^#^*p* < 0.05, ^##^*p* < 0.01 comparison between S50 group, S100 group, and S150 group.

To further investigate the effect of HA NPs on osteogenic differentiation of hMSCs, the expression of ALP, OPN, Runx2, and OCN was assessed using RT-PCR after 14 days of induction (Figure 7). In agreement with the ALP activity and immunofluorescent staining results, HA NPs increased the expression of all four bone-related genes. For the cells incubated with of S50 and S100, the expression levels of ALP, OPN, Runx2, and OCN all displayed significant increases compared with that of S150 group.

### 3.5. Calcium Ion Release

**Figure 8:**
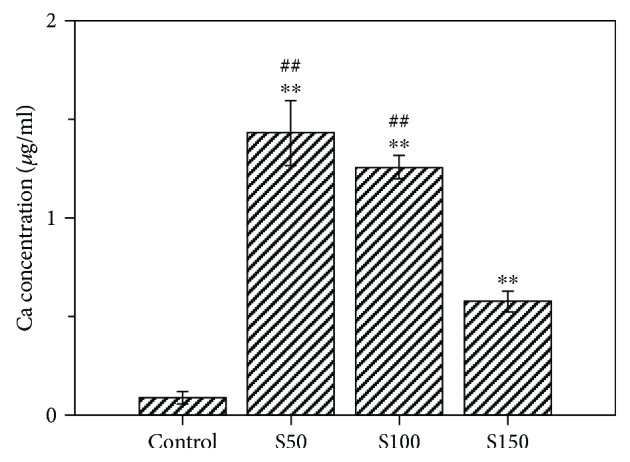
Release of calcium ions from 10 *μ*g/ml of S50, S100, and S150 in DPBS at 37°C for 7 days. Values are expressed as mean ± SD (*n* = 3 for each sample). ^∗∗^*p* < 0.01 compared to the control group (hMSCs without HA NP treatment). ^##^*p* < 0.01 comparison between S50 group, S100 group, and S150 group.

The release of calcium ion (Ca^2+^) form HA NPs in DPBS was investigated using ICP-OES. As shown in Figure 8, Ca^2+^ concentrations of S50, S100, and S150 groups were significantly higher than that of the control group (DPBS only). Moreover, after 14 days of soaking, the Ca^2+^ release form S50 and S100 showed a significant increment of 147% and 117% as compared with that of S150. The result indicates that HA NPs of all sizes possess degradability and smaller-sized HA NPs (S50 and S100) appear to degrade faster than larger-sized S150.

### 3.6. The Effect of HA NPs on Medium pH

After 2 days of incubation under standard cell culture condition, the pH of the control group (medium without HA NPs) was 7.23 ± 0.02, while the pH of medium with S50, S100, and S150 was 7.23 ± 0.02, 7.24 ± 0.01, and 7.26 ± 0.02, respectively, all displaying no significant differences compared with the control group. The result indicates that the addition of HA NPs does not change the pH of culture medium.

## 4. Discussion

HA NPs have been widely used for applications in the field of biomedicine and tissue engineering [8, 32], such as hard tissue repair, biomedical imaging, and drug delivery. These applications associated with HA NPs require that they have different shapes and sizes. In this study, HA NPs with three different sizes were prepared by chemical precipitation method via altering the temperature and pH of reaction solution (Figure 1). Calcium ions and phosphate anions firstly formed amorphous calcium phosphate (CaP) or hydrated orthophosphates, which can subsequently transform to HA through phase transformation at suitable conditions [33]. Thus, the growth process of HA was prolonged and slow [34]. Higher precipitation temperature, supplying higher amounts of energy, allowed HA NPs grow faster. The diffraction peaks S50 and S100 were wide and low, while S150 showed well-differentiated peaks (Figure 2), which may be caused by the increased c-axis of the unit cells of S150 under higher precipitation temperature. Murakoshi et al. also prepared different-sized hexagonal CdS nanoparticles through varying the preparation temperature [35]. In addition, due to the different pH value, crystallite facets of HA NPs absorbed different amounts of OH^−^ [36]. The shielding effect of OH^−^ on the interface could control the growth rate of the OH^−^-absorbed crystallite facets [37]. Taken together, the different sizes and aspect ratios of S50, S100, and S150 were caused by altering pH value and precipitation temperatures.

Cytocompatibility assessment result in this study showed that S50, S100, and S150 were cytotoxic to hMSCs in a dose-dependent manner. Many previous researches have already reported the dose-dependent cytotoxicity of HA NPs to several cell types through inducing necrosis or apoptosis [1, 23, 24, 38, 39]. The degree of cell death caused by HA NPs had a strong correlation with the amount of particle load [23, 38]. The HA NPs were degraded in cell lysosomes to increase intracellular Ca^2+^ concentrations, which might cause lysosomal rupture to induce cell necrosis [40]. On the other hand, the anticipated agglomeration and subsequent sedimentation of HA NPs at relative high concentrations might result in mechanical damage to the cells, which could cause cytotoxicity [1, 41]. These reasons may be possible mechanisms for the concentration-dependent cytotoxicity of S50, S100, and S150 to hMSCs.

Previous researches involving HA NPs and osteogenesis mainly focused on the effect of coatings or scaffolds doped with HA NPs on osteogenic differentiation of bone-related cells [18, 20, 21]. For instance, Wang et al. found that the hydroxyapatite nanoparticles/polyamide (HA NPs/PA) composite scaffolds had no negative effects on the adhesion, proliferation, and osteogenic differentiation of rabbit bone marrow-derived mesenchymal stem cells (rBMSCs) [18]. In contrast, this study investigated the *in vitro uptake* of HA NPs with different sizes and their influence on differentiation of hMSCs into osteoblastic phenotype. A major finding of the present study is that S50, S100, and S150 have stimulatory effect on osteogenic differentiation of hMSCs reflected by increased ALP activity and enhanced expression of bone-related markers (Figures 5–7). ALP is an early marker during the osteogenic differentiation, which can provide phosphate groups for subsequent hydroxyapatite deposition [42]. Runx2 acts as the initial and most specific marker that can activate and regulate osteogenic differentiation [43]. OCN, synthesized only by fully differentiated osteoblasts, is a specific marker of mature osteoblasts [44]. OPN, which can enhance mineralization by its calcium and collagen-binding properties, is also a marker of osteoblasts [45]. Similar results have been found in previous studies, where HA NPs showed positive effects on osteogenesis [25, 46, 47]. For instance, HA NPs with a diameter of about 20 nm have been reported to promote the type I collagen, OCN, and OPN expressions of rabbit mesenchymal stem cells [25]. Moreover, Wang et al. prepared HA nanospheres (~50 nm in diameter) and HA nanorods (~50 nm in length) through the induction of protein template and found that both nanoparticles could significantly enhance the osteoblastic differentiation of rat mesenchymal stem cells, especially the HA nanospheres [46]. The enhancement of osteogenic differentiation of hMSCs may be related to the changes of culture medium condition caused by HA NPs [25]. HA NP-conditioned medium (obtained by soaking HA NPs in culture medium for 3 days and centrifuging to remove HA NPs) has been proven to promote the osteogenic differentiation of bone marrow-derived mesenchymal stem cells [48]. Small shifts in extracellular pH could result in significant changes in the ability of hMSCs to express markers of the osteoblast phenotype [49]. The activity of human osteoblasts has been proven to increase with the increasing medium pH during the range from 7.0 to 7.6 [49–51]. It is known that the original pH of culture medium of most cells varies in the range of 7.2–7.4 due to the physiological pH. In this study, after 2 days of incubation, the pH of culture medium with and without HA NPs was comparable during the range of 7.2–7.3. The addition of HA NPs did not change the medium pH. Therefore, the positive effects of HA NPs on osteogenesis may be related to other condition changes of culture medium, such as the concentration of calcium ions, caused by HA NPs rather than the medium pH.

Another major finding is that S50 and S150 appear to have stronger role in stimulating the osteogenic differentiation of hMSCs than S150. The cells treated with S50 and S100 expressed more ALP, OPN, and Runx2 compared with that treated with S150 (Figures 5–7). Similar results could be found in a previous study, where rat bone marrow-derived mesenchymal stem cells (rBMSCs) expressed higher levels of osteoblast-related markers by the stimulation of smaller HA NPs than that of larger ones [33]. The size of HA NPs is an important factor for affecting the biological behavior of bone-related cells [32, 52, 53]. Smaller-sized HA NPs may change the microenvironments of cell culture which can greatly enhance osteogenesis [54]. Smaller-sized HA NPs adsorb proteins forming a neomatrix different with that formed by larger-sized HA, which can greatly impact osteogenesis [33]. Calcium ions (Ca^2+^) have been shown to affect the growth and osteogenic differentiation of stem cells [55, 56]. Greater concentrations of Ca^2+^ significantly increased the extent of cell mineralization [56]. Ca^2+^ plays an essential role in maintaining the cell growth and functions [57]. Futhermore, Ca^2+^ can activate MAPK signaling pathway, which is important for inducing cell differentiation [58, 59]. S50 and S100 released more than two times of Ca^2+^ as S150 after 14 days of soaking in DPBS (Figure 8). The larger specific surface area and aggregation size of S150 (Table 2) may result in its lower degradation speed. Due to the differences in degradation speed, smaller-sized HA NPs (S50 and S100) release higher concentration of Ca^2+^ than larger-sized HA NPs (S150), which may result in their different effects on osteogenic differentiation of stem cells. These may be the reasons why the smaller-sized HA NPs show greater stimulatory effect on osteogenic differentiation of stem cells.

## 5. Conclusions

In this study, HA NPs with three sizes (S50, S100, and S150) were prepared. All the data demonstrated that HA NPs of all sizes had stimulatory effect on the osteogenic differentiation of hMSCs *in vitro*. The hMSCs incubated with smaller-sized HA NPs (S50 and S100) seemed to have higher differentiation rate compared with that treated with S150, indicating that the efficiency of osteogenic differentiation of hMSCs was dependent on the size of HA NPs. This difference may be caused by the different concentrations of Ca^2+^ released by S50, S100, and S150. These suggest that the size of nanoparticles is an important factor needed for consideration in biological environment and will provide a reference for HA NPs in biomedical applications.

## Figures and Tables

**Table 1:** Primers used for RT-PCR study.

Gene	Primer (5′ → 3′)
ALP	F: AGCACTCCCACTTCATCTGGAA
	R: GAGACCCAATAGGTAGTCCACATTG
OPN	F: CTCAGGCCAGTTGCAGCC
	R: CAAAAGCAAATCACTGCAATTCTC
Runx2	F: GCCTTCAAGGTGGTAGCCC
	R: CGTTACCCGCCATGACAGTA
OCN	F: CACTCCTCGCCCTATTGGC
	R: CCCTCCTGCTTGGACACAAAG
GAPDH	F: GAAGGTGAAGGTCGGAGTC
	R: GAAGATGGTGATGGGATTTC

**Table 2:** Hydrodynamic diameter and surface area of HA NPs. Values are expressed as mean ± SD (*n* = 3 for each sample).

Particle	Hydrodynamic diameter (nm)	Surface area (m^2^/g)
S50	568 ± 20	63.30 ± 0.94
S100	626 ± 15	66.73 ± 0.95
S150	1262 ± 47	46.80 ± 1.19

## Data Availability

The data used to support the findings of this study are available from the corresponding author upon request.
